# Climate change: Comparing “green” and “polluting” nation-states

**DOI:** 10.3389/fsoc.2023.1133333

**Published:** 2023-02-22

**Authors:** Lorenzo Posocco, John R. McNeill

**Affiliations:** ^1^School of Sociology, University College Dublin (UCD), Dublin, Ireland; ^2^Department of History, Georgetown University, Washington, DC, United States

**Keywords:** climate change, China, Russia, nationalism, green nationalism, United States of America (USA), sustainability, environmentalism

## Abstract

Some nation-states, i.e., Norway, Sweden, and Denmark, repeatedly score the highest in environmental indicators such as the Environmental Performance Index (EPI) and the Climate Change Performance Index (CCPI). Their cities win environmental awards; they have well-developed recycling systems; they perform well with biodegradable waste; and their citizens show awareness of environmental problems, protesting publicly and even sueing their governing bodies if they don't do the same. For these and other reasons, recent scholarship defined these countries as “exemplary” green nation-states. The question is, which factors pushed them toward the green transition faster than others? And overall, what stops top polluting countries such as China, the United States and Russia from walking the same path? This article attempts to answer these questions by looking at climate change through a theoretical framework based on theories of nationalism and case studies of green nation-states. It compares three of said top polluting countries, China, the United States, and Russia, with “exemplary” green nation-states, and argues that the pace of greener nation-states rests on (1) a tradition of ecologism and environmentalism rooted in the long run, (2) the lock in of “green nationalism,” a form of nationalism grounded on sustainability, (3) free and effective environmental movements, (4) inclusivity and welfare, and (5) a sense of national pride in environmental achievements. The available evidence seems to suggest that top polluting nation-states lack one or more of these factors.

## 1. Introduction

**Figure 1:**
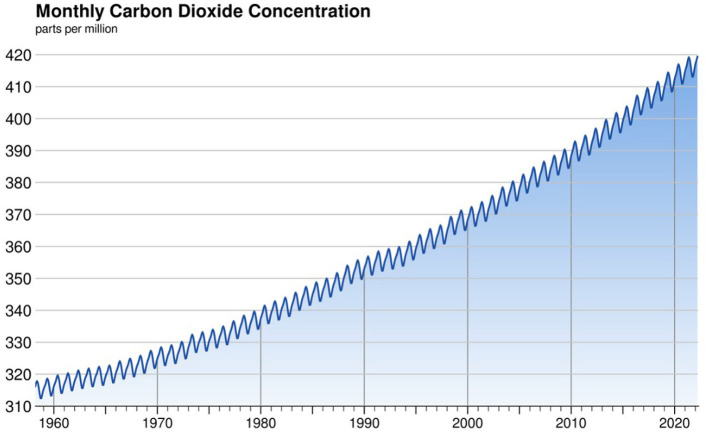
CO_2_ concentrations over the past 60 years. Available online at: https://scrippsco2.ucsd.edu/graphics_gallery/mauna_loa_record/mauna_loa_record_color.html. Graph is in the public domain and free to use (accessed May 07, 2022).

The COVID-19 pandemic raised awareness about planetary-scale risks. In January 2020, a global pandemic didn't even make it into the top 10 of the most likely risks identified by the World Economic Forum's Global Risks Report 2020 (Goldin, 2021). Despite repeated warnings from the scientific community, governments worldwide didn't believe it was a serious possibility. Today, after it has killed more than 6 million people across the globe (WHO, 2022), the spectrum of what seems possible has widened. As a result, more attention is paid to other such risks, including atomic conflicts, financial crises, deadlier pandemics, and climate change. Especially the latter, which is likely to be the greatest threat to human and non-human life on earth (Conversi, 2020), is discussed in yearly summits, such as the recent COP27, reports published by intergovernmental organizations such as Nobel Prize winner IPCC (International Panel for Climate Change), and warnings launched by the UN (2022). The question is, if a single virus traveling our globalized world could do what it did, what will happen when temperatures rise? When extreme heat waves bring drought and crop failure? When dengue fever and malaria expand their domains? When rising sea levels imperils low-lying coastal communities (Colón-González et al., 2021)? Vis-à-vis this possibility, science suggests that it is paramount to keep the earth's temperature from rising 1.5°C above pre-industrial levels, and avoid potentially catastrophic scenarios (IPCCB, 2021). There is scientific agreement that to keep such levels, we must stop the use of fossil fuels that release carbon dioxide (CO_2_) and other greenhouse gases (GHG) into the atmosphere (Crutzen and Brauch, 2016) (see Figure 1 below representing the increase in CO_2_ since 1960). These are the main culprits for global warming, wrapping the earth like a blanket and trapping the sun's heat.

One of the effects of the pandemic and its lockdowns has been to decrease CO_2_ emissions. In fact, media and social media feasted on it, reporting on transport fleets forced to the ground, the price of oil halving worldwide, resurgent wildlife with hardly any humans outdoors, and rapid declines in air pollution. For all their faults, media have raised general awareness about climate change, especially by spreading the powerful message that the exploitation of nature doesn't come without consequences. And yet, increasing visibility and awareness didn't result in an immediate change of course with respect to climate change. The European Union's Copernicus satellites brought evidence that by March 2021 air pollution in Europe had already returned to pre-pandemic levels (Copernicus, 2021). A study by Sarmadi et al. (2021), which analyzed air quality index variations before and after the onset of the COVID-19 pandemic in 87 cities around the world found the same result: improvement was temporary. While a number of announcements —including the return of the USA to the Paris Agreement and pledges made for the COP26 meetings in Glasgow in late 2021 (e.g., President Xi Jinping's promises that China will reach peak emissions before 2030 and become carbon neutral by 2060; India's plan to meet 50% of its energy requirements from renewable energy by 2030)—bring hope, similar statements by heads of states in the past induce skepticism. Global governance has so far failed to tackle global risks (Goldin, 2013), and that includes both pandemics and climate change, and, pledges notwithstanding, there is no strong evidence that things will be different in the near future.

The problem is not the science. We have climate solutions that could be implemented right now and make a difference (Harvard Center for Climate, Health, and the Global Environment, 2021). We have a number of prominent studies that propose seemingly optimal solutions and complex models involving numerous variables. But we don't know how to make sure that those in power do what is required. That is why most recent models, such as Cullenward and Victor (2020), identify politics as the main problem and attempt to suggest solutions. And yet, all these models ‘are highly divergent in the factors they think matter’ (Cullenward and Victor, 2020, p. 13). What's new in this article is not attention to politics. Rather it is the identification of new factors that change the way we look at climate solutions.

Some of these factors were recently identified by a small body of studies that have started to tackle the politics of climate change through theories of nations and nationalism (Conversi, 2020, 2022; Conversi and Posocco, 2022; Posocco and Watson, 2022a,b). The heuristic value of these studies is evident in the fact that the world is divided into nation-states, which are the building blocks of all international organizations and international agreements (Conversi and Posocco, 2022). If these agreements do not bring the desired fruits it is because national governments do not implement climate plans (even when they agree to them) decided at international summits, i.e., The United Nations Conference on Environment and Development (Rio de Janeiro, 1992), The Kyoto Protocol (1998), The Cancun Agreement (2010), the Paris Agreement (2015), etc. These agreements are never legally binding, and breaking them comes without notable consequences for powerful nation-states such as the United States of America (Chomsky and Pollin, 2020). For example, Donald Trump's administration withdrew the USA from the 2015 Paris agreement. During a speech at the White House where he claimed that reducing carbon emissions would cost Americans jobs, Trump said: “I was elected to represent the citizens of Pittsburgh, not Paris. I promised I would exit or renegotiate any deal which fails to serve America's interests” (BBC, 2018). And that was it. With a nationalist speech, the US was out (until 2021).

It is true, today more than ever “we inhabit a global village” (Goldin, 2021, p. 172), but the nation-state remains “the dominant political reality of our time” (Brubaker, 2015, p. 115), and it is driven by nationalism: the “dominant mode of political legitimacy and collective subjectivity in the modern era” (Maleševic, 2019, p. 17). Proposing solutions for climate change without coming to terms with the nation-state and nationalism means losing sight of the protagonist in politics. And yet, no study before 2020 addressed climate change from the perspective of theories of nationalism (Posocco and Watson, 2022b). Before 2020, the geo-historical notion of Anthropocene, which identifies an era where human beings have the biggest impact on the environment, “did not appear in any of the issues of the main journals dedicated to nationalism studies (*Nations and Nationalism, Ethnicities, Nationalism and Ethnic Politics, Ethnopolitics, SEN—Studies in Ethnicity and Nationalism, Nationalities Papers*” (Conversi and Posocco, 2022, p. 2).

This article's goal is to add more research to the interrelations between nationalism, climate change, and its resolution. To do so, it builds on a theoretical framework highlighting five factors shared by what we define as “green nation-states”' driving Green Nationalism (GN), a form of nationalism that is based on environmentalism and sustainability. These are: (1) a tradition of ecologism and environmentalism rooted in the long run, (2) the spreading of environmentalism at the national level and its lock in within the institution of the nation-state, (3) free and effective environmental movements, (4) inclusivity and welfare, and (5) a sense of national pride in environmental achievements. These factors will function as guidelines through which this article looks at three top carbon polluters; China, Russia, and the USA, and compares their environmental trajectories with Sweden, Norway, Denmark, Switzerland, and Germany, countries that have often been ranked at the highest levels of two of the most important environmental indexes: the Environmental Performance Index (EPI), and The Climate Change Performance Index (CCPI).^1^

## 2. Theoretical framework

**Figure 2:**
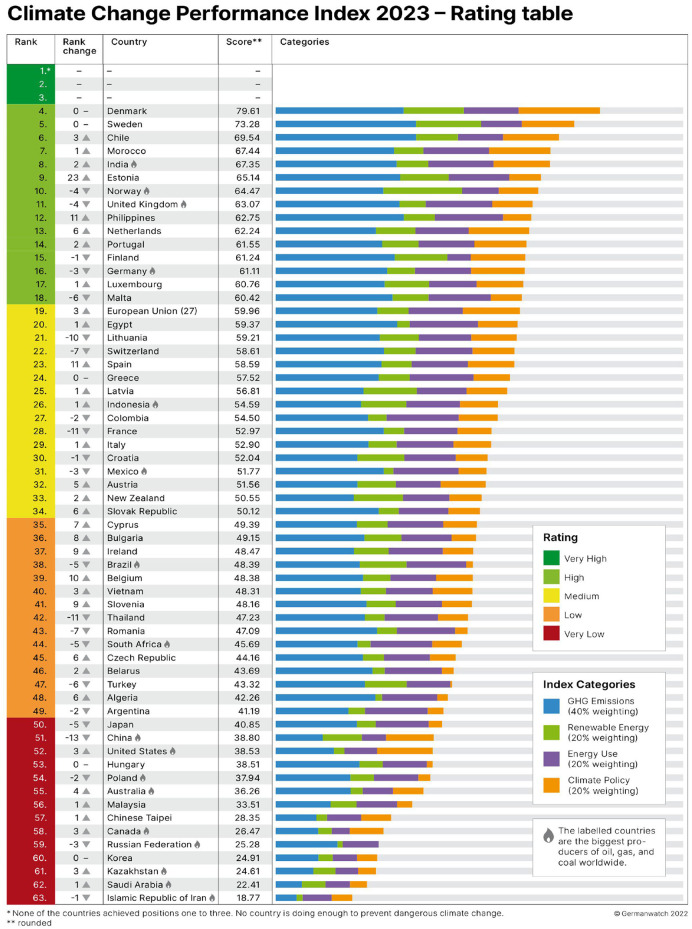
CCPI ranking 2023.

**Figure 3:**
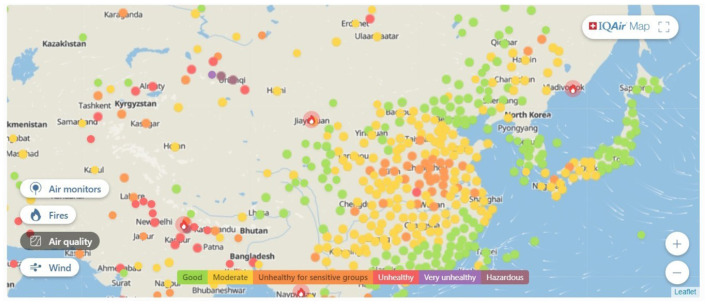
Air quality in China by IQAir.com. Red means “unhealthy” (data recorded on 02/02/2022).

The pace at which most nation-states implement reforms to address the climate crisis is as slow as it is worrisome, but some are slower than others. While it is true that even in those nation-states that sit at the top of CCPI rankings (see Figure 2) there is much room for improvement. Countries that have been identified as green nation-states (Conversi and Posocco, 2022; Posocco and Watson, 2022b), among them Denmark, Sweden, and Norway are the top performers, and Germany as a relatively high performer, are better placed than others to function as examples to follow. Some cities in these countries have clear goals in place, i.e., Copenhagen aims to be the world's first carbon-neutral capital city by 2025 and Oslo will probably reach net zero emissions by 2030, which is much more than what others are trying to achieve. According to a recent study, Freiburg is also close to this goal (Umweltbericht, 2021). Should other cities in China, the US, and Russia follow the example of Oslo and Freiburg, the world could avoid the climate crisis' worst-case scenarios recently outlined by Kemp et al. (2022). Instead, most cities lag behind. For example, in China, only 1 in over 52 cities (Figure 3 below) has pollution levels below the recommended limits. GHG emissions in China surpass those of the entire developed world combined (Rhodium, 2021).

Recent studies on green nation-states suggest that the building blocks of these societies' environmental “success” lie in (1) a tradition of ecologism and environmentalism rooted in the long run, (2) the lock in of Green Nationalism, (3) free and effective environmental movements, (4) inclusivity and welfare, and (5) a sense of national pride in environmental achievements (Conversi and Posocco, 2022; Posocco and Watson, 2022a). The first point is supported by evidence that environmentalism in green nation-states owes its vitality to environmental ideas and practices strongly entrenched in their communities. These ideas came upon the national scene first in the nineteenth century, as a reaction to the negatives of the industrial revolution, including pollution, hard life and working conditions. In these countries, the search for alternatives started earlier than, say, China, where the disastrous effects of industrial modernity on the environment have only recently been acknowledged. In green nation-states alternatives took many forms, among which *friluftsliv* (“free air life”) in the Scandinavian regions, and *Lebensreform* (“life reform”) in Germany and Switzerland (Conversi and Posocco, 2022). They represented proto-ideological guidelines in terms of the human-nature relationship. The shift in outlook created an alternative to the idea of nature as something to exploit: nature as enriching the human experience on earth, a treasure to protect and safeguard for future generations. These ideas formed the core of a counter cultural phenomenon, especially in a time when industrial capitalism exploited nature at an unprecedented speed. For example, when Ibsen wrote his ecological *Paa Vidderne*, Karl Marx was working on *Das Kapital* (published in 1869). Both must be seen as reactions to modern industrialization and capitalism, and their problematic relationship with the social but also the natural world. Both could be considered as the first attempts at creating knowledge against the unintended side-effects of modernity (Beck, 1992, 2016).

Indeed, movements that proposed ideological alternatives to the single-minded exploitation of nature can be found in most green nation-states. They were soon accompanied by the creation of numerous institutions raising awareness about nature as something to protect against human exploitation. In Scandinavia, these ideas spread through institutions such as *Fältbiologerna* or *Friluftsfrämjandet* (Swedish Outdoor Organization) in Sweden, The Danish Outdoor Council (*Friluftsråde*), and The Norwegian Trekking Association (*Den norske turistforening*; Kaijser and Heidenblad, 2018), in Germany the German Alpine Club (*Deutscher Alpenverein*). These (and many other such) institutions have been important vehicles through which a more ecological mindset spread through the nation, instilling a number of ideas including that harming nature equals to wrongdoing.

There is no room here for outlining the many environmental movements and organizations of green nation-states, both historically and in the present. We leave it to the already mentioned emerging literature on Green Nationalism, conscious that a more thorough review of these studies is needed to deepen said connections. What's important is to highlight that these movements were the building blocks of environmental ideas crystallizing over several generations, culminating in a more robust tradition of national environmentalism in comparison to other nation-states (Conversi and Posocco, 2022; Posocco and Watson, 2022a). These ideas, stored in the closets of these nations' collective memory (Halbwach, 1950[1980]) and embraced by the political institutions, prepared the ground for environmental movements that, in the 1960 and 1970's, were the backbone of historical protests against environmental destruction such as Earth Day (1970). These movements pushed governments to act, draft and enact environmental policy that were unthinkable just a few years earlier, and found fertile grounds in their nation-states' administrations.

That said, traditions of environmental ideals and environmental activism that is strong and overall free are two factors that characterize greener nation-states driven by Green Nationalism, but they are not the only ones. In green nation-states, environmental policy and sustainable practices follow as elements that determine the passage from environmental ideas to environmental practices. Most importantly, environmental policy in these nation-states came from the embrace of environmentalism by the political institutions of these nation-states (Conversi and Posocco, 2022). This is a crucial passage from environmentalism to Green Nationalism (Posocco and Watson, 2022b), which could be defined as the institutionalization of environmentalism materializing in national ministries, ministers, policies and regulations. That equals to the lock in of environmentalism, which means that the ideas stemming from the environmental ideology becomes entrenched in social and political systems that help spread it further (McNeill, 2000). If this process lasts, environmentalism becomes a “doxa” (Bourdieu, 1992; Wacquant, 1995). Deeper rooted than any orthodoxy or heterodoxy: doxic beliefs aren't even discussed. They are taken for granted.

A green transition stretching across a nation needs the pervasive bureaucracy of the state, its organizational structure coordinating a vast network of state institutions, a national judicial system that sets environmental laws and a national police that enforces it. Margulies (2021) stated that “to achieve ecological sustainability and preserve natural ecosystems, political responses must transcend the boundaries of national borders, and forge a system of international cooperation built on the collective enforcement of international environmental agreements” (Margulies, 2021, p. 28). Indeed, while international environmental agreements are important, change is, for structural and organizational reasons, always implemented by the nation-state at the national level. In this view, the nation-state and nationalism can be (and are) positive forces (Maleševic, 2013; Harari, 2019; Tamir, 2020) that are already playing a decisive role against climate change. A state entrenched in Green Nationalism is strongly instrumental to reverse unsustainable trends, turn polluted cities into ecological points of reference, and give birth to environmental trends that have significant echoes at the local and national level, and even beyond.^2^

There are many studies supporting this notion and focusing on, for example, Vauban (Buehler and Pucher, 2011; Fraker, 2013; Daseking, 2015), Zurich (Theurillat and Crevoisier, 2012), or at the nation-state level, Sweden, Denmark, and Germany (Anderberg and Clark, 2013; Uekotter, 2014) providing strong evidence of politics championing environmentalism as a major force pushing, say, businesses to comply with environmental regulations or individuals to act responsibly toward the environment. Championing environmentalism also increases pride at the local, regional and national level, which is a key factor, another incentive for nation-states to undertake the green transition and political parties to leverage it (Posocco and Watson, 2022a).

For the sake of clarity, it is true that at the local level some “exemplary green” nation-states might look to be strongholds of environmentalism while at the national level they carry out business as usual. But it would be wrong to see the local and the national as two distinctive and compartmentalized entities. They are not. More often than not, when it comes to environmental funding, regions and cities largely benefit from various forms of national funds [i.e., even in Italy, which falls behind in terms of green transition, the recently established Ministry for the Ecological Transition redirects national and European funding to regions and cities (Ministero della Transizione Ecologica, 2022)]. In most green nation-states, national governments grant cities and regions with the freedom and the resources they need to pursue environmental aspirations. A good illustration would be the adoption of Local Agenda 21^3^ by the Danish government, which made it compulsory by law within its territory, loading municipalities with more responsibility (but also more room for maneuver and decisional power; Nordic Report, 2018). Moreover, state policy often echoes local realities. For example, green nation-states grant the rights of citizens everywhere within their large national territories to exercise voice and influence on which services are provided and how they are delivered. This leads to consider the importance of inclusivity and welfare.

Most exemplary green nation-states are “actively inclusive states” (Dryzek et al., 2002). They don't only listen to and accept the demands of environmental activists, turning them into state policy, but secure a “desired pattern of interest articulation” (Dryzek et al., 2002, p. 660). And they are strong welfare states. Research shows that there is a link between welfare regimes introduced at the state level and positive attitudes toward climate policy at the local level (Sivonen and Kukkonen, 2021), especially when it comes to eco-taxes. The nexus is rather logical; better life conditions (i.e., national health care, better salaries, free education, etc.) create more trust in governments that can more easily pass laws such as eco-taxes that are usually very unpopular.

## 3. Top polluting nation-states: What went wrong?

### 3.1. United States of America

As mentioned in the previous section, five factors seem to play an important role in the greening of nation-states: (1) tradition of environmentalism, (2) grassroots environmental activism, (3) environmental policy sustained in the long run, (4) sustainable practices at various levels of society, and (5) bonding between environmentalism and nationalism leading to national pride in environmental achievements. When considering the US, it is evident that it doesn't lack a tradition of environmentalism (Harris, 1984; Rovinskaya, 2017). Indeed, this tradition goes as far back in history as those of Germany, Switzerland, and Scandinavian countries, and, at times, shaped the US into one of the most progressive nation-states in terms of environmentalism. As in exemplary green nation-states, the first environmental movements were born in the US as a consequence of and reaction to massive industrialization in the nineteenth century and the relentless exploitation of natural resources (Stewart, 2015). If Denmark had Henrik Ibsen, and Germany had Herman Hesse, the US ecological movement is linked to the figures of John Muir^4^ and Henry David Thoreau (among others) who functioned as real environmentalists ante litteram.

**Figure 4:**
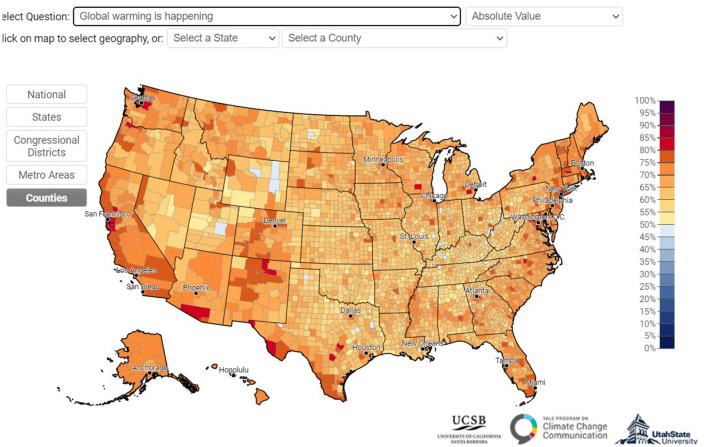
Yale climate opinion maps. Available online at: https://climatecommunication.yale.edu/visualizations-data/ycom-us/ (accessed December 14, 2022).

Environmentalism in the US has its greatest stronghold on the West Coast, in particular in the San Francisco area, where the Sierra Club, one of the most important environmental organizations in the US (and in the world), was founded in the nineteenth century. Given the importance of environmental tradition, one of the points this article stresses, it is not surprising that San Francisco repeatedly comes in first place in environmental indexes (such as the “Green City Index” sponsored by Siemens that compares cities in the US and Canada). The Yale Climate Opinion Maps (2021), which show Americans' climate change beliefs, risk perceptions, and environmental behavior, also suggests variation between states in the US (see Figure 4 below).

**Figure 5:**
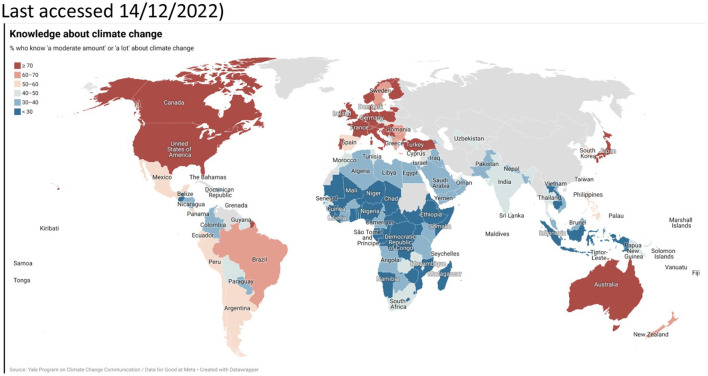
International public opinion on climate change, 2022. Available online at: https://climatecommunication.yale.edu/publications/international-public-opinion-on-climate-change-2022/toc/3/ (accessed December 15, 2022).

**Figure 6:**
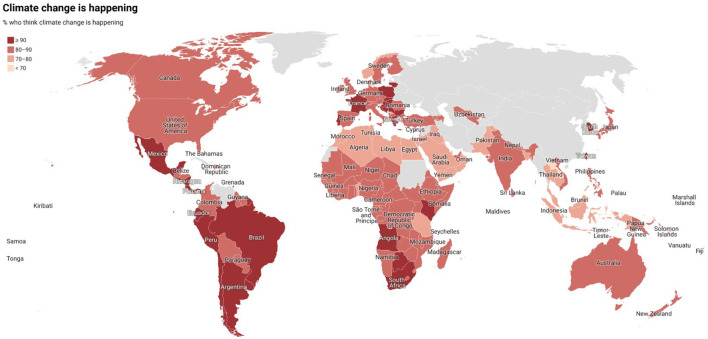
International public opinion on climate change, 2022. Available online at: https://climatecommunication.yale.edu/publications/international-public-opinion-on-climate-change-2022/toc/3/ (accessed December 15, 2022).

It doesn't mean that environmental knowledge and awareness in other American regions is necessarily less rich. The 2021 International Public Opinion on Climate Change report suggests that Americans think to know about climate change as much as the citizens of green nation-states (see Figure 5). Like most of these nation-states, between 70 and 80% of Americans believe that climate change is happening (see Figure 6).

**Figure 7:**
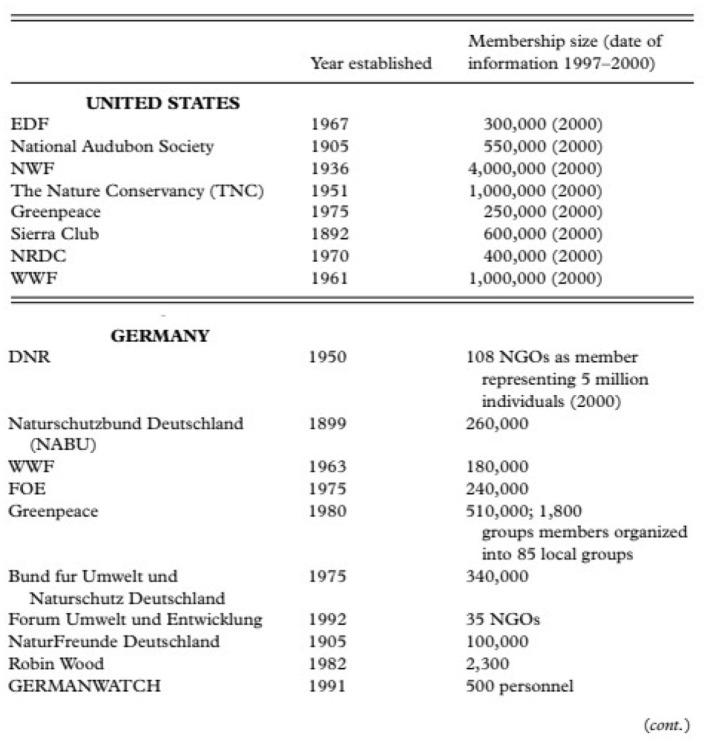
Comparison between US and German environmental NGOs. US environmental NGOs are much bigger than Germany's. The study from which this figure derives (Schreuer's “Environmental Politics in Japan, Germany and the United States,” in bibliography) emphasizes that there are many environmental groups in the US with budgets in the millions, while similar groups in Germany are much poorer. Although it is important to highlight that at the beginning of the new millennium, the population of the US was 3.5 times bigger than Germany's.

As for environmental regulations, they existed in the US as far back as the ones of Germany and the other exemplary green nation-states we focus on. Similarly to green nation-states, regulations in the nineteenth century in the USA didn't aim to protect nature from exploitation but to increase the efficiency of extractions vis-à-vis the growth of industry (Stewart, 2015, p. 142). Environmentalism, environmental justice, and environmental policy are phenomena mainly tied to the late 1960's, when they also emerged in the most progressive nation-states in Western Europe [in 1970–75 the Environmental Protection Agency (EPA) was founded and numerous environmental laws were passed]. Finally, the size and richness of US environmental NGOs is good evidence of the vigor of the environmental movement in the US (see Figure 7 below).

The fact that the US has a strong tradition of environmentalism, environmental activism, and environmental awareness, suggests that this country meets two over five factors mentioned above that project nation-states onto a greener path. Where does the US stand when it comes to the remaining factors, the share of environmentalism by the political institutions, inclusivity, welfare, and environmental pride?

Unlike exemplary green nation-states, US politics is strongly entrenched in lobbying. Large investor-owned fossil fuel producers such as ExxonMobil (among others) use lobbying to minimize disruption since the strengthening of environmentalism in the 1970's and the growing pressure of environmental NGOs (Kenner and Heede, 2021). For obvious reasons, they have enormous interests in keeping things the way they are, and are willing to spend liberally to influence governments (Klein, 2019; Chomsky and Pollin, 2020). Fossil fuel producers and other magnates of industry are especially determined to prevent governments from enacting laws that would, i.e., tax the disastrous environmental effects of their operations or halt mining and drilling activities. Such measures alone could oblige top polluting companies to either make their activities greener or shut down. That's where lobbying comes in. Corporations perform lobbying in various ways; Baumgartner et al. (2009) list inside advocacy (personal contacts politicians, dissemination of external research to policy-makers, etc.), outside advocacy (public relations campaigns, paid ads, etc.), and grassroots advocacy (mobilizing mass membership, organizing a lobby day, etc.) as attempt to hinder governmental action that would undermine their polluting activities.

For the sake of clarity, lobbying and other forms of corporate political activity also occur in exemplary green nation-states, but there are differences between the latter and the US. The most important was highlighted by Mahoney (2007), who said that the US system fails to reach compromise nearly 75% of the time. It is a system where the winners take it all, and especially when direct elections couple with private campaign finance and lobbying, the outcome is biased in favor of wealthier business interests. In the EU, industry wins too but so do citizen groups and foundations.

Another problem is that the US is entrenched in neoliberalism at a level that green nation-states are not. Neoliberalism as a variant of classical liberalism is based on the idea that people should be given maximum freedom to pursue their self-interest. This clashes with most of the exemplary green nation-states in this study, where the market is subject, more than in the US, to state regulation. In a neoliberal system, governments are less prone to intervene in the economy and regulate the activities of gargantuan corporations that end up overpowering them and/or influencing them significantly. The situation in the US worsened when, in 2010, the United States Supreme Court in Citizens United v. Federal Election Commission overturned a long-standing precedent regarding the First Amendment rights of corporations and permitted unregulated direct political spending by corporations in election campaigns. As a result, elected politicians find themselves in the very difficult position of owing their office to big polluters while they are publicly expected to put a stop to their operations.

This hinders governmental actions that might (and do in green nation-states) make a difference. One among many, carbon taxes (Cullenward and Victor, 2020). If government taxed polluting activities, and rendered them unsustainable while promoting green technology and making it available through state incentives, pollution would inevitably drop. This happened in Norway where eco-taxes are very high and provide the basis for this country's path to CO_2_ reduction (Ostli et al., 2021).

The USA's reluctance to “making climate policy work” (the reference here is to Cullenward's and Victor's work on climate policy, 2021) couple with a form of capitalism that sociologist Matthew Desmond defined as nothing less than “brutal” (Diamond, 2019). This form of capitalism, states Desmond, is inherently more exploitative than others of people and environment. In particular, it creates a high degree of class inequality (Eppard et al., 2018; Kumar, 2018). These are all factors not consistent with green nation-states driven by green forms of nationalism, most of which are actively inclusive, organizational, and welfare states that make equality one of their primary goals. For the sake of clarity, the correlation that this article makes between welfare and climate change performance is not causal. We don't mean that implementing welfare will automatically improve climate change performance. However, existing studies suggest, as mentioned in the previous section, that a number of positives come from welfare in terms of environmental action. In particular, welfare states enable every citizen, even the most disadvantaged, to fully participate in the political life of their countries. Since there is strong evidence that “the most severe harms from climate change fall disproportionately upon underserved communities who are least able to prepare for, and recover from, heat waves, poor air quality, flooding, and other impacts” (EPA, 2021, p. 1), it is from these segments of society that one expects demands for radical change. It is fair to suggest that feeding inequality and injustice, “brutal” capitalism also gives birth to the conditions that keep the status quo. In this perspective, green nationalism and forms of extremely exploitative capitalism are poles apart; two ideologies that can hardly coexist. This is something highlighted already by both Klein (2019) and Chomsky and Pollin (2020). That said, it is not surprising that most green nation-states are also social democracies. They are capitalist in terms of economy but maintain similar values to socialism. Their politics play, more than in the US, the fundamental role to balance economic and social needs.

Finally, an element that works against the US, but also China and Russia, in building a green nation-state, is their sheer size and complexity. It is true that developing a unified environmental ideology at the national level might be a greater challenge for these countries than smaller ones such as Denmark or Sweden. But it is also true that bigger nation-states such as Germany, that shares the same political system as the US—like the US, Germany is a federal state—as well as many problems, including but not limited to historical economic and ideological differences between Länder or the UK, have decreased their carbon emissions steadily since the 1990's. (1) Bigger population could be an issue. It equals higher energy consumption and higher emissions. The US has four times the population of Germany, and one might think that comparing the emissions of these two countries is unwise. And yet, a simple equation shows that if we multiply Germany's population four times and calculate their energy consumption, Germany would still be consuming around 2 milliom GW·h/yr less than the US [according to data from the World Bank and The U.S. Energy Information Administration (EIA)]. (2) The reality is, the average electrical power per capita in Germany is around half as much as that in the US. Indeed, there wouldn't be any problem in consuming energy if its source came, as in Norway or Sweden (whose energy consumption is twice that of the US) and increasingly also Germany, from renewable sources, where greenhouse gas emissions associated with stationary energy use would be low. But this is not the case in the US, whose energy needs is covered principally by natural gas (31.8%), petroleum (crude oil and natural gas plant liquids 28%), coal (20%), and nuclear power (9,6%) while only 12.7% comes from renewables (official data from the U.S. Energy Information Administration [EIA]).

### 3.2. Soviet Union and Russia

Before the late 1980's, the Soviet Union didn't leave much space, if at all, to environmental organizations and grassroots environmental activism. For the sake of clarity, many such organizations and individuals proliferated in Tsarist Russia and under Lenin's rule for the same reason as they proliferated in Europe and the US; pushed by the evidence that the natural world was being annihilated by modern industrialization. However, unlike in the US and green nation-states, they disappeared or were silenced after Stalin took over (Josephson, 2013). Among the scientists that greatly contributed to the idea of natural conservation were Vasily Dokuchaev, Mikhailovich Knipovich, and Grigory Kozhevnikov. In particular, Kozhevnikov is commonly known for having conceived *zapovedniks*; protected areas that go beyond the concept of John Muir's national park by being completely free from human interference. Among the environmental organizations, the All-Russian Society for the Protection of Nature was born in 1924 and became the most influential voluntary society devoted mainly to nature protection (Josephson, 2013). It carried out incredible work in terms of education and research. Yet, like in most of the other modern states in the nineteenth and the first half of the twentieth century, their studies were mainly used by the state for improving exploitation rather than conservation (McNeill and Engelke, 2016). Among the Russian intellectuals that functioned as environmentalist ante litteram were well-known novelist Fyodor Dostoevskii, Fedorov, and Bulgakov, whose work reflected the need to defend nature from uncontrolled exploitation. These writers saw natural sites as inextricably linked to the nation-state, a tangible and natural part of it (Ely, 2002).

Interestingly, before Stalin, “a variety of scientific and other public actors pushed civil initiatives and established voluntary associations, amateur societies and movements aimed at nature and culture protection” (Josephson, 2013, p. 107). Under Stalin, they basically stopped functioning. First of all, Stalin inaugurated an era of technocratic euphoria where the environment was either an enemy or a resource to exploit, a concept very much in line with Resource Nationalism. The situation remained unaltered under Khrushchev (Mazhitova et al., 2021). Most importantly, Soviet authorities saw all independent organizations, including environmental ones, as potentially treacherous organizations hatching anti-Soviet ideals and/or bearers of national separatism. They were violently eliminated through arrests, exile and other methods. The result is that Stalinists destroyed the country's pre-revolutionary environmental memory, which in turn resulted in much weaker environmental movements and activism when compared to green nation-states or even the US.

After decades of environmental immobility vis-à-vis the massive exploitation of Soviet territories, the Chernobyl disaster (1986) functioned as a real crossroads that loosened the already weakening Soviet grasp on its satellite countries and triggered a series of environmental protests. Often called “eco-nationalisms” (Dawson, 1996; Malloy, 2009; Perga, 2021), these environmental movements took shape especially in Soviet satellites: Ukraine, Belarus, Armenia, and in the Baltic countries. The latter erupted when it was clear that Moscow not only exploited these territories but endangered their populations and ecosystems, and systematically lied about it (Yaroshinskaia, 2006). In this context, environmentalism was a drive toward greater autonomy and self-determination. Some even argued that it was determinant to the fall of the Soviet Union (Dawson, 1996).

The massive protests that followed Chernobyl, coupled with Gorbachev's push toward democratization, were two phenomena that paved the way for the birth of environmental organizations in many Soviet and post-Soviet states, including Russia. After the fall of the Soviet Union, Russian NGOs were still alive, but had no support from the state that was struggling to pick up the pieces. This era of Boris Yeltsin's rule (1991–1999), Henry (2010) called the time of “benign neglect” during which the Russian state neither supported nor hindered the work of environmental NGOs. Financial need led many of these NGOs to lean on foreign funding, a process that also influenced their internal structure and made them more akin to Western-style institutions (Evans, 2006).

**Figure 8:**
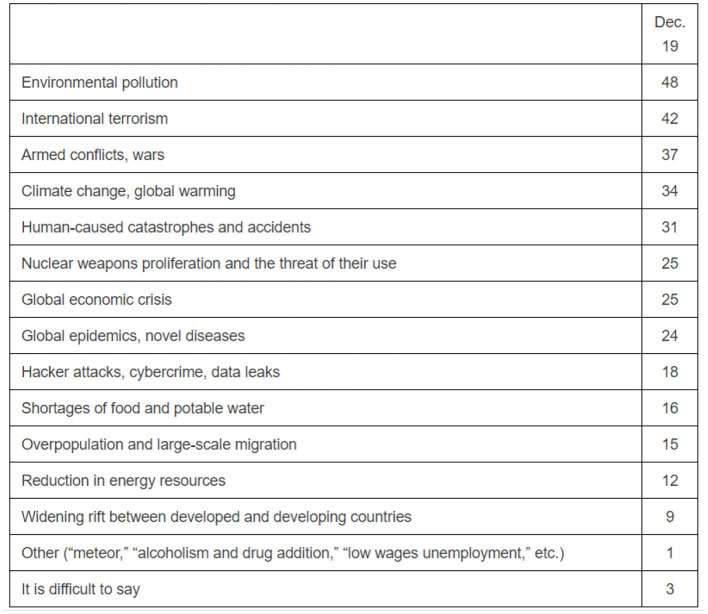
Levada-Center, From Opinion to Understanding, Environmental problems in Russia. Russians believe that environmental pollution is a major threat to their lives and put climate change and global warming at the 4th place after terrorism, 2nd place, and war, 3rd. Available online at: https://www.levada.ru/en/2020/02/18/environmental-problems/ (accessed February 23, 2022).

This short trend lasted around a decade. It ended with the rise of Vladimir Putin after 2000, whose administrations increasingly excluded NGOs from governance, and after 2010, repressed them (Plantan, 2018). The year 2010 saw the biggest (and perhaps unparalleled) environmental protest in Russian history, organized to safeguard the Khimki Forest in Moscow. This protest, which tackled the construction of a highway through one of the Moscow region's last remaining woodlands, should be seen as the fruit of environmentalism after around 15 years of activism in Russia (its origins, as mentioned above, are to be found earlier in the Soviet Union). Just as it happened in the US and exemplary green nation-states in the 1970 and 1980's, people in Russia who were exposed to the principles of environmentalism started to mobilize around protection of the environment in the 2000's. Environmental awareness among Russians today (see Figure 8 below) should be seen as the product of this initial exposure to environmentalist principles.

**Figure 9:**
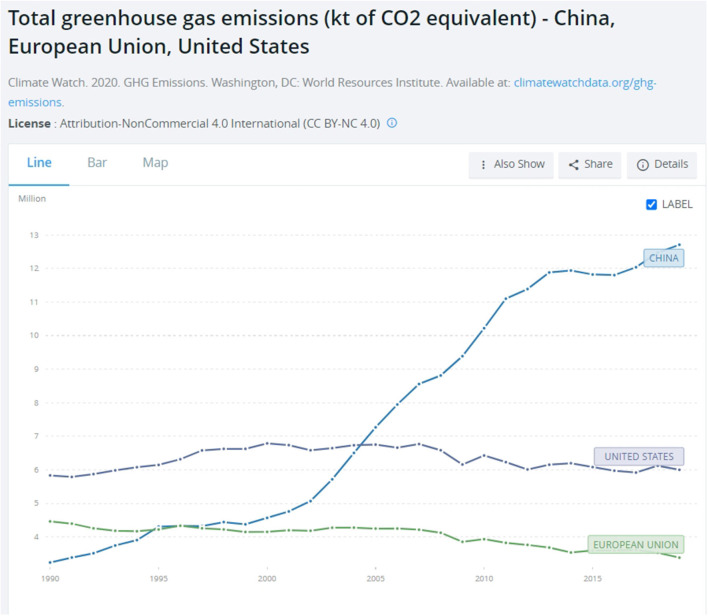
CO_2_ emissions of China, the US and EU. Evidence from the World Bank, available online at: https://data.worldbank.org/indicator/EN.ATM.GHGT.KT.CE?locations=CN-EU-US (accessed February 06, 2023).

As of today, Russia remains strongly entrenched in Resource Nationalism and a green transition is not yet on the horizon. This country is ranked fourth in the world in primary energy consumption and carbon dioxide emissions (Mitrova and Melnikov, 2019). In fact, the latter increased in recent years (see Figure 9 below). Unlike green nation-states, governments kept a skeptical attitude toward climate change. This coupled with the quasi total unsuccess of environmental movements vis-à-vis an authoritarian regime that today, and yesterday, attempts to capitalize on “muscular” rather than “green” nationalism. The ongoing conflict in Ukraine, which brought destruction and crises on all fronts, humanitarian and environmental, and Crimea, Transnistria, South Ossetia, and Abkhazia before that, is proof of this. It also suggests that environmentalism never locked in in Russia as it did in green nation-states. Environmental policy was never sustained in the long run, which is another important element in green nation-states.

Finally, in terms of inclusivity, democracy, and welfare, Russia performs worse than the US, which was not up to the standard of green nation-states. The The Global Wealth Report (2021) states that 110 Russian citizens control over 35% of total household wealth across the country, “most of which is connected with natural resources such as gas and oil” (Posocco and Watson, 2022a, p. 8). According to the Europen Parliamentary Research Service (2022), wealth inequality has increased over the past two decades, a fact that is supported by Voeykov and Anisimova (2022, p. 729), who state that “economic inequality in Russian society has reached a critical line fraught with serious social threats.”

### 3.3. China

China's industrial modernization occurred late in comparison to powers such as the US and Russia, and so did independent Chinese environmental movements, born to fight against the side-effects of said modernization. Friends of Nature was the first NGO registered at the State Ministry of Civil Affairs in China on March 31st, 1994 (Yang, 2005). Environmental history studies also developed only recently, in the second half of the 1990's (Bao, 2004). It was the quick deterioration of the environment that “urgently demanded academic research to provide the necessary and latest knowledge for treatment” (Bao, 2004, p. 476).

China's carbon emissions skyrocketed in the last two decades, providing it the unenviable title of top carbon emitter (see Figure 9 below). China is also in 51st position of 64 countries in the Climate Change Performance Index (CCPI, 2022), receiving an overall very low rating (see Figure 2 above). This means that it is doing very little to reduce those emissions and fight climate change. At the same time, research shows that China is significantly affected by changing weather patterns (World Meteorological Association, 2022). Its temperature warmed, over a period of 70 years, at a much higher rate than the global average. As a result, it witnessed a growing number of extreme weather events, especially heavy rainstorms causing flooding (Zou et al., 2021).

Initially, concerned by a regime that repressed public protests (sometimes with extreme violence as in the 1989 massacres at Tiananmen Square), independent environmental organizations played “safely” by focusing on education and protection of biodiversity. However, they soon started implementing other forms of pressures such as carefully organized public demonstrations, lawsuits, and used social media to openly discuss pollution and other environmental problems (Balme, 2014). Most of the time, independent environmental organizations did so by maintaining an attitude of caution, conducting campaigns that would “only” implicitly challenge the government policy or criticize the government for failing to enforce environmental protection laws (Stalley and Dongning, 2006).

Protests increased after the country joined the World Trade Organization (WTO), especially when the private sector started to grow, and due to China's meteoric industrialization, the demand for raw materials and energy increased steadily. The disruptions associated with mining, for coal and metals, and with giant-scale hydroelectric dam-building, aroused popular resistance [coal still supplies around 58% of China's energy (National Bureau of Statistics of China, 2020)]. Environmental activists organized demonstrations against the Three Gorges Dam, the Dams on the Nu River, Tiger Leaping Gorge, and the 2012 “Shifang protest” in the city of Shifang, Sichuan province, against the building of a copper plant (Wang, 2013; Steinhardt and Wu, 2016). Yet, the real outcomes of these protests are uncertain. Some studies posit that the increased number of protests might “stand at the forefront of broader changes in the landscape of Chinese sociopolitical activism and contentious politics” (Steinhardt and Wu, 2016, p. 61) while others stress that protests weren't effective, that in most cases the regime didn't step back, and that it kept (and will keep) environmental organizations in check, monitoring their activity, controlling the formation of civic groups, and limiting group gatherings (Lin, 2007). Perhaps both things are happening at the same time, as China developed a model of “participatory authoritarianism” (Owen, 2020) based on extending civic participation while maintaining strong control over it. This was a governance strategy pioneered in the 1920's by Italian fascists and Soviet apparatchiks. In such a framework, NGOs are allowed to work and demonstrators are allowed to protest within certain limits established by the regime.

These are all elements that show how different China is from green nation-states whose pace in terms of environmental performance is based, among others, on a longer tradition of environmentalism and environmental activism, and free environmental movements that inform the state and from which the state is informed. These factors, which seem to be important preconditions to the development of green nationalism as an ideology shared by large sectors of society, represent something that China is far from achieving. How does China perform when it comes to the remaining two factors highlighted above, inclusivity and welfare?

**Figure 10:**
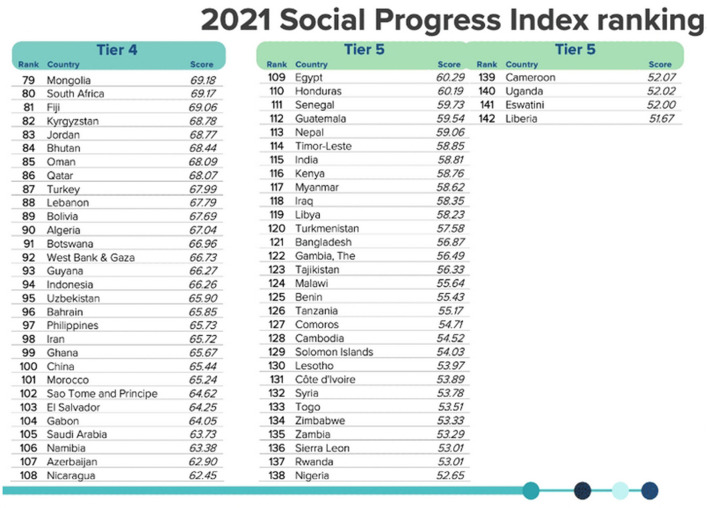
The 2021 Social Progress Index Rankings (SPI) places China in 100th position out of 168 countries. Available online at: https://www.socialprogress.org/?code=CHN (accessed February 06, 2023).

**Figure 11:**
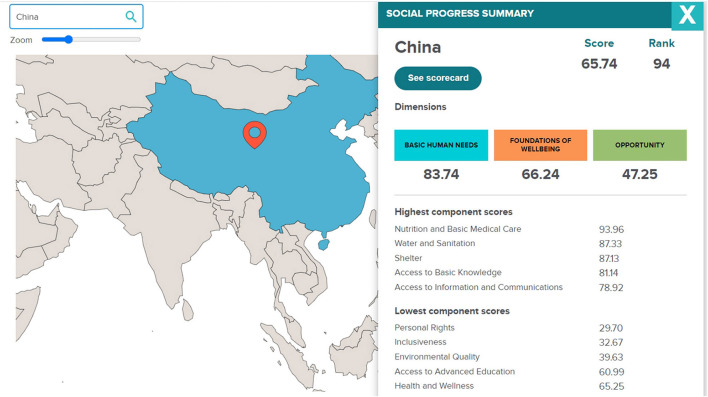
The 2022 Social Progress Index Rankings reveals China's weakness in both inclusiveness and opportunity. Available online at: https://www.socialprogress.org/?code=CHN (accessed February 06, 2023).

The 2021 Social Progress Index Rankings (SPI), which is based on the work of Amartya Sen, Douglass North, and Joseph Stiglitz, and measures the wellbeing of a society by observing social and environmental outcomes, places China in 100th position out of 168 countries (see Figure 10 below). Interestingly, top positions in this ranking are occupied by green nation-states, a fact that brings further support to the argument that a strict correlation exists between welfare and climate change performance. Regarding China, the SPI reveals this country's weakness in both inclusiveness and opportunity (see Figure 11 below). In terms of opportunity, China doesn't ensure freedom of peaceful assembly, access to justice (including environmental justice), freedom of discussion, and basic political rights to its citizens, all elements that are at the core of green nation-states. In terms of inclusiveness, unlike green nation-states, China doesn't do enough to ensure that no one is excluded from the opportunity to be a contributing member of their society.

**Figure 12:**
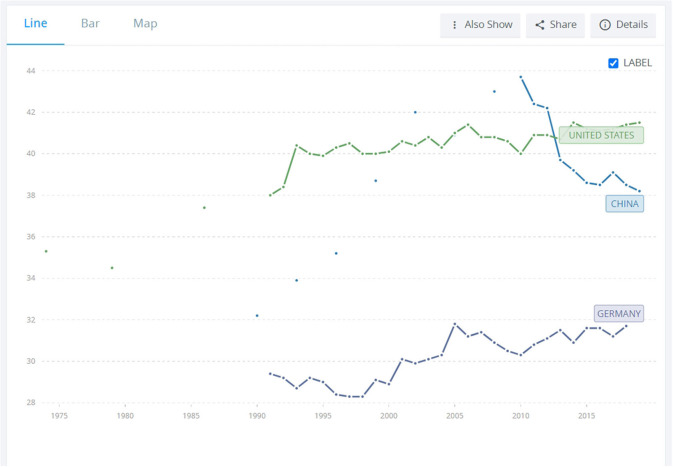
GINI index, comparison between US, China and Germany. Available online at: https://data.worldbank.org/indicator/SI.POV.GINI?locations=CN-US-DE (accessed February 06, 2023).

In terms of poverty and inequality, the GINI index, which measures the statistical dispersion representing the wealth inequality within a nation, places China in between the US and Germany (see Figure 12 below). This is due to the fact that, as Figure 12 shows, poverty levels in China dropped substantially in the last 10 years, an achievement reached under President Xi Jin Ping's administration (Yang and Liu, 2021). China became the world's largest developing country to eradicate extreme poverty in 2020, placing itself higher than developed countries, i.e., the US. While China is far from performing as a green nation-state (in the GINI index, only 3 points separate China from the US, while there is an 11 point difference between China and Norway, which is the best performer among green nation-states), recent developments in this country show improvement.

An element that these indexes do not consider is the shift toward the global market, coupled with low-cost of manufacture and impressive technological progress, which made China the main producer, exporter and installer of green technology in the world (IRENA, 2020). This includes solar panels, wind turbines, batteries and electric vehicles, placing this country at the forefront of the global energy transition. Chinese wind turbine manufacturers account, impressively, for one-third of global supply (Helveston and Nahm, 2019). China is also the worldwide market leader in electric vehicles (Statista Report, 2021) with sales amounted to over 834,000 in 2019—nearly three times greater than the combined sales of the leading markets in Europe. Considering that transportation represents an important part of CO_2_ emissions (around 14% according to IPCC 2014), positive developments in this sphere bring hope. In fact, green technology innovation can greatly reduce the environmental effects arising from fossil energy consumption (Wang et al., 2021), especially when green technology locks in, which seems to be happening in China. As Conversi and Posocco (2022) put it, green technology plays a major role in green nation-states, and China seems to be at the forefront of it. However, these are all very recent developments, and while China's climate and energy policy might be at a turning point (Heggelund, 2021), to follow in the footsteps of green nation-states, this country should fill the gaps in terms of inclusiveness, freedom of expression, and opportunity.

## 4. Conclusion

In this article, the issue under scrutiny is the factors that projected some nation-states toward the green transition much faster than others, and what stopped (and keeps stopping) top polluting nation-states such as China, the United States and Russia from walking the same path. It developed an original theoretical framework based on theories of nationalism and case studies of exemplary green nation-states that scored the highest in environmental indexes, especially CCPI and EPI, and outlined five factors at the core of their environmental performance: (1) the development of a tradition of ecologism and environmentalism rooted in the long run, (2) the lock in of green nationalism across society, (3) free and effective environmental movements, (4) inclusivity and welfare, and (5) a sense of national pride in environmental achievements.

This article introduced the hypothesis, that a tradition of environmentalism, overlooked by previous studies, might be a key element in green nation-states to ensure that environmental values, beliefs, and habits lock in, planting the seeds across society, including the political sphere. It also considers that the capacity of environmental groups to express dissent if governments' policies do not tackle the climate crisis is a fundamental power in the hands of civil society in green nation-states. The possibility to make their voice heard and counterbalance political and economic interests seems to be an important factor characterizing green nation-states. Additionally, in these nation-states, environmental movements go hand in hand with inclusivity and welfare. Inclusive forms of state favor the exercise of voice and influence from various sectors of society, especially disadvantaged ones. Finally, being seen as the world champions of the environment is an important element adding to these countries' national pride and giving birth to a virtuous circle that fuels both.

One or more of these elements are lacking in the three top polluting nation-states that this article investigated: China, the US, and Russia. First of all, for logical reasons, none of them developed pride in environmental success. Regarding a tradition of environmentalism among top polluting nation-states, the US has the oldest and strongest environmental tradition, but its political system is entrenched, more than green nation-states, in (1) lobbying, a feature that favors big polluting companies with money to burn, (2) neoliberalism, and (3) a form of capitalism that fosters inequality. Unlike green nation-states, which are all inclusive states, the US can be considered an exclusive state in the sense that the voices of those who have more resources receive far more attention than others in the United States Congress and every state's legislature. The result is that US environmental policy is extremely responsive to forces that have the resources to and the interest in keeping things from changing. On the contrary, green nation-states tend to be “stronger” states, not in the military or economic sense, but in that they tend not to leave social and environmental problems to markets and corporations. In addition, these countries' welfare systems ensure much lower gaps in terms of wealth distribution between poor and rich. This is key insofar as economic inequality in the US keeps large segments of society out of the political life of the country. As a result, those who are affected the most by climate change, namely the poor and disadvantaged, are too busy making ends meet to successfully express their dissent.

Powerful centralized states such as Russia and China, are not better placed than the US to host the changes that a fast green transition requires. Both suffer from chronic forms of authoritarianism that, despite heroic citizen efforts, leaves little space for environmentalism, therefore hindering the development of green forms of nationalism. As a result, environmental ideology hasn't locked in as it did in exemplary green nation-states. Environmental values and beliefs aren't as widespread as they should be to (a) mobilize segments of the population that could perform various forms of environmental action, including protests and awareness-raising campaigns, (b) push governments to consider environmental problems and enact policy aimed to solve them, and (c) generate long-lasting environmental habits among the population.

It is difficult to say whether these countries could go beyond the limits imposed by their political systems, and perhaps do what China is trying to do, which is lead a green transition that is top-down and centralized, relying more on green technology whose spreading (at home and abroad) is aided by China's low-cost of manufacture. In this scenario, the greening of China hasn't much to do with civil society, inclusivity, welfare, and citizens' participation but with a government that has invested heavily in green technology and provided momentous subsidies and incentives to the sector (Finamore, 2020). The same top-down green transition is probably not possible in Russia, a country that, so far, hasn't exhibited the same economic dynamism, and it is more involved in muscular rather than green nationalism, as proven by its recent and less recent interventions in Ukraine, Crimea, Transnistria, South Ossetia, and Abkhazia.

Finally, this article acknowledged that the larger political economic picture puts countries such as Norway and Denmark on a different footing from China, Russia, and the US. The sheer size and complexity of these countries can (and probably does) work against the development of a more unified environmentalist stance. In addition, the history of these countries and their (perceived) geopolitical imperatives might play a role in hindering stronger responses to climate change. These are all factors adding to those mentioned above, working against top polluters' capacity to re-modernize and become greener versions of themselves.

## Data availability statement

The original contributions presented in the study are included in the article/supplementary material, further inquiries can be directed to the corresponding author.

## Author contributions

All authors listed have made a substantial, direct, and intellectual contribution to the work and approved it for publication.

## Footnotes

^1^CCPI link: https://ccpi.org/; EPI link: https://epi.yale.edu/.

^2^See the many studies of green nation-states: Vauban (see Buehler and Pucher, 2011; Fraker, 2013; Daseking, 2015), Zurich, in Switzerland (Theurillat and Crevoisier, 2012), or Sweden and Denmark (Anderberg and Clark, 2013).

^3^Local Agenda 21 is a non-binding action plan of the United Nations with regard to sustainable development. It originated in Rio de Janeiro at the 1992 United Nations led Hearth Summit and adopted by 178 governments.

^4^The story goes that Muir's book “Our National Parks,” brought him to the attention of President Theodore Roosevelt. “In 1903, Roosevelt toured the American West, and requested the opportunity to camp with Muir in Yosemite Park. There, together, beneath the trees, they laid the foundation of Teddy Roosevelt's innovative and notable conservation programs. After that meeting with Muir, Roosevelt embarked on a course of action that established 148 million acres of National Forest, five National Parks and 23 National Monuments during his term of office” (Sierra Club, 2022). Donald Worster (A Passion for Nature: The Life of John Muir, Oxford University Press 2008), says 230 million acres were set aside for federal protection during Roosevelt's tenure in office.
